# Ed Nickoloff, Sc.D. Obituary by Michael D. Mills

**DOI:** 10.1002/acm2.12695

**Published:** 2019-08-08

**Authors:** Michael D. Mills

**Affiliations:** ^1^ Radiation Oncology University of Louisville Louisville KY USA

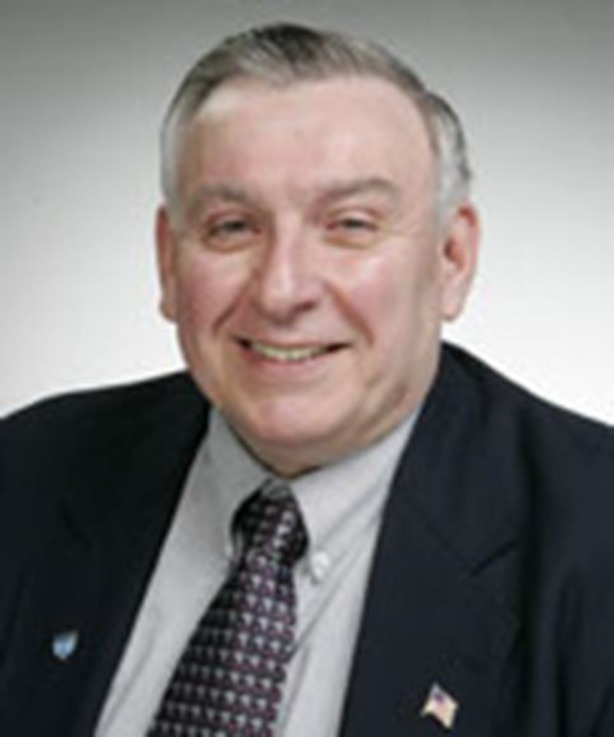

Dr. Nickoloff, from Orangeburg, New York passed away on 11 March 2019 after a long illness. He was born in Harrisburg, Pennsylvania, and grew up just outside of Harrisburg in the suburbs between Harrisburg and Hershey. He grew up where the air smelled like chocolate all the time. He grew up in a land of dairy farms and orchards. He was active in sports, and his recreation included hiking, gardening, automobile mechanics, and reading.

Ed had fond memories of family picnics in Hershey, and summer vacations at the beach in Atlantic City, NJ. On Ed’s father’s side, the family originated from the country of Macedonia, which is north of Greece in the mountainous region. Macedonia was one of the former Yugoslav Republics. Alexander the Great, who conquered most of the known world was from Macedonia. Ed loved that Mother Teresa was also Macedonian. Throughout his schooling, Ed excelled, making mostly straight A’s all the way through elementary, junior high and high school. Ultimately, he graduated second in his high school class of 434 students. Ed elected to attend Carnegie Institute of Technology, which is now known as Carnegie Mellon University. However, at the end of Ed’s sophomore year, his mother Pearl became very ill. To be closer to home, Ed transferred to Lebanon Valley College to finish his bachelor’s degree. At Lebanon Valley College, Ed made the Dean’s list all of the time, and the Chairman of Physics went to bat for him to receive several Fellowships. That made it possible for Ed to attend graduate school after receiving his B.S. degree, which he did in 1965.

Ed chose to attend the University of New Hampshire, because of the scenic beauty of the campus. Ed’s Master’s thesis project was thermal neutron detection with pulsed shaped discrimination using liquid scintillation detectors. After receiving his Master of Science in 1968, Ed went to work briefly at the Massachusetts Institute of Technology Research Laboratory of Electronics, where he worked in the electronics industry.

Shortly afterward, Ed’s mother died of cancer, and his father and brother (now 13 yr old) needed him. Ed returned to Harrisburg, and taught high school for 1 yr, and later returned to the electronics industry, working for AMP Semiconductor Electronics. However, his mother's death due to cancer refocused the direction for his life, and after a few years, he went to Johns Hopkins University. There he did research in early Nuclear Medicine heart studies, working on the prototype for what we now call Multi‐gated Acquisition or MUGA studies. There he worked with some of the giants of Radiology, Henry N. Wagner, Jr., Bertram Pitt, William Strauss and Philip Alderson.

For his Sc.D., Ed did research in the early Nuclear Medicine heart studies. After his thesis defense, the examination committee made him worry in the hallway for about 30 min while they celebrated with champagne; then, they finally invited him in for a glass of champagne and told him he graduated with a special honor—“with Distinction.”

Ed was asked to teach the Radiology Residents at Johns Hopkins. Evidently, he made a good impression because he was offered the position permanently and made Acting Director of Physics and Engineering. He stayed at Johns Hopkins in Radiology another 8 yr.

One day, Dr. Philip Alderson relocated to Columbia University, and invited Ed to come along. This was the big city, full of concrete, culture, museums, Broadway theater, symphony, opera, restaurants, and so much more. Now Ed would be free to release his cosmopolitan soul and search among the most elegant, refined and cultured ladies in the world for that special someone.

When Ed met Diane, she was a social worker (MSW degree) at the Lavelle School for the Blind with a special interest of working with children. Later, Ed inspired her to pursue additional medical education, and she eventually received a BSN degree and an RN certificate. She was very compassionate with people and truly cared for them. She also had many traditional values about hard work, honesty, and respect which Ed admired. She also wanted to get married and have a family. Ed dated her a number of times and he proposed to Diane—the traditional way on one knee. They were engaged and married within 14 months; a testimony to Ed’s usual decisiveness. Today, Diane is a Certified School Nurse in New Jersey and continues her life’s calling to look after young people with health needs.

In 2003, Ed, Jim Astarita and I came up with a way to make the JACMP work. Ed was ACMP Chairman elect at the time and was willing to take the risk to back this new way of publishing, and commit the JACMP to being a true open source, open access journal. The JACMP would not be here today but for Ed. We all owe him thanks, and I most of all!

Ed could fix, repair or replace anything from asphalt to zoysia grass. He loved to garden with the children until he watched an array of animals chew it all up. He fought back by digging a trench, building a cinderblock wall and topping that with a 4‐foot fence. His solutions are a little "over the top." However, his efforts were not in vain. That year, the garden yielded a bumper crop of tomatoes and a 9‐pound zucchini. Those ground hogs were no match for Ed.

He was at hockey games at 4:00 AM on Saturdays (this is not a typo—these games were at 4 AM!) and Sunday football, cheerleading, fishing, sledding, PTA, church related activities, hiking, and occasional short weekends away. He was an avid reader, Jack of all trades, and outdoorsman. Ed was well‐read and could speak on any topic whether it be history, philosophy (especially his own), and of course every science. Education was paramount and when the children were in college, they did not just call for money. They will call to ask questions about their academic subjects. They often asked their mother, "How does dad know all that?" Ed is survived by his wife of 35 years, Diane (Zambetti); daughter, Andrea; son Edward Jr., and daughter‐in‐law, Katarina.

Ed Nickoloff was an Emeritus Professor of Radiology at the Columbia University College of Physicians & Surgeons and Chief Hospital Physicist at the Columbia University Medical Center for 33 years. During his time at Columbia, Ed built an imaging physics section to provide those resources needed to meet the hospital’s demanding requirements. Of particular note are the members of his staff who stayed with Ed for decades, an excellent indication of his positive management and personal styles.

Ed lectured extensively at scientific conferences across the country, wrote two books on the subject of Radiation Physics, one book used extensively in Radiology Residency programs across the USA, published 150 journal articles, 57 peer‐reviewed journal articles, and 87 abstracts. His writing style was inclusive; typically, everyone who he asked for information or help was included as an author on the resulting publication. In addition to everything else, Ed held 24 offices in professional organizations.

Furthermore, Ed was definitely the alpha dog of the small town of Orangeburg. Ed was fair‐minded and honest almost to a fault. As honored and as modest as he was about his many awards and achievements, I am sure he would agree that his greatest accomplishment was his family. He deserves to be proud of both. Finally, Ed also knew how to separate work from his personal life and focus on life outside of physics when that's where he was. This was the way he conducted himself throughout his career, always being a man of high principle and always a true gentleman. He truly was a credit to our profession. He will be greatly missed.

I thank Steven Balter and Diane Nickoloff for their informed and insightful contributions. I also thank Associate Editors‐in‐Chief Timothy Solberg and Per Halvorsen for their valuable and perceptive comments.

